# Vitamin E-coated membrane reversed itchy rash during hemodialysis

**DOI:** 10.1007/s40620-024-01941-8

**Published:** 2024-05-02

**Authors:** Fulvia Zappulo, Miriam Di Nunzio, Gabriele Donati, Anna Scrivo, Anna Laura Croci Chiocchini, Gaetano La Manna

**Affiliations:** 1 Department of Medical and Surgical Sciences (DIMEC), Alma Mater Studiorum—University of Bologna, Bologna, Italy; 2 Nephrology, Dialysis and Kidney Transplant Unit, IRCCS–Azienda Ospedaliero-Universitaria di Bologna, Via Massarenti, 9, 40138 Bologna, Italy; 3 Nephrology, Dialysis and Kidney Transplant Unit, Azienda Ospedaliero-Universitaria di Modena, Modena, Italy; 4 Surgical, Medical and Dental Department of Morphological Sciences, Section of Nephrology, University of Modena and Reggio Emilia, Modena, Italy

**Keywords:** Dialyzer, Hypersensitivity, Vitamin E, Itchy rash

## The case

**Fig. 1:**
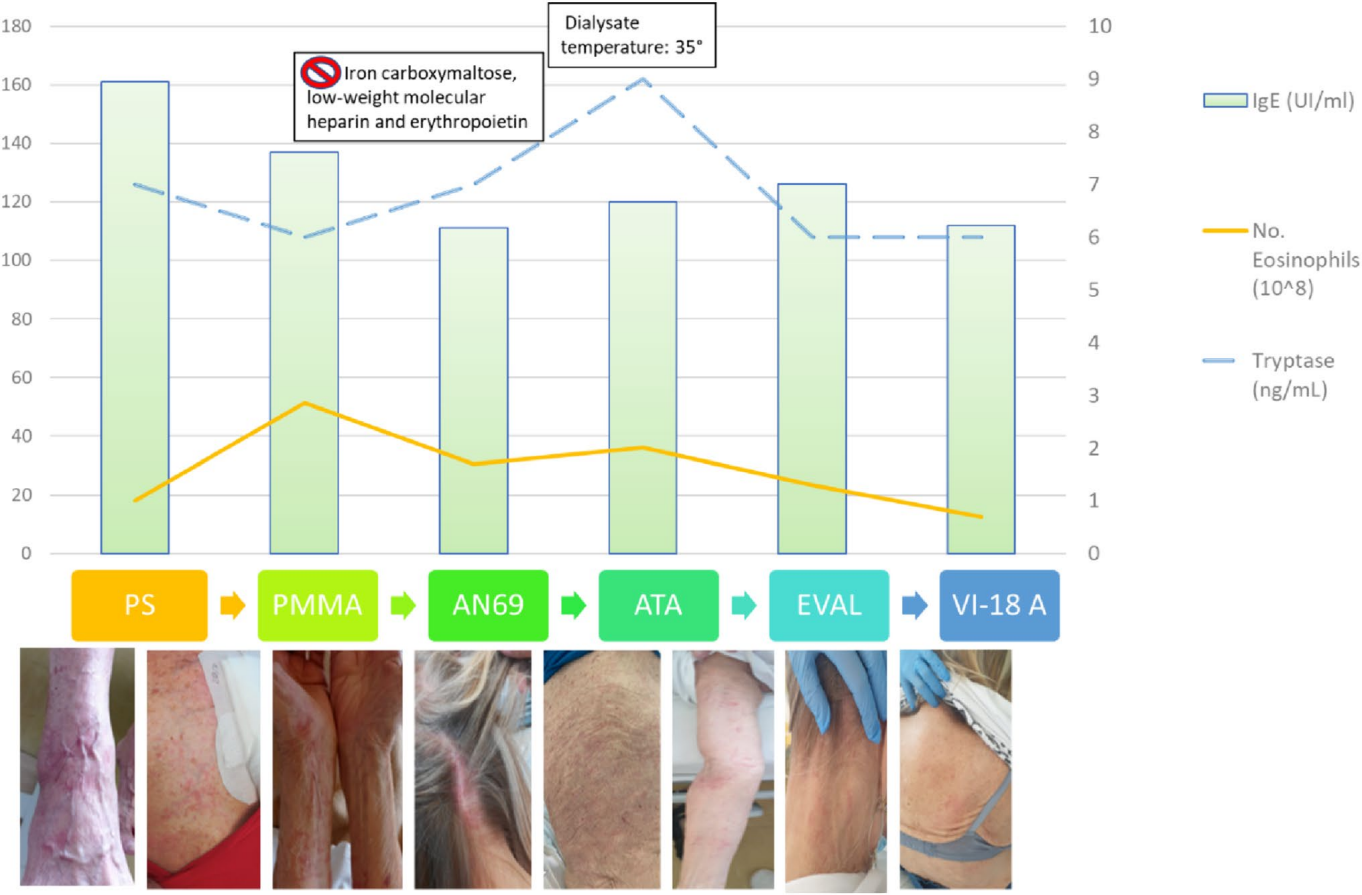
Skin rash during hemodialysis. This figure represents the evolution of the skin rash that occurred during the dialysis sessions with different dialyzers and the trend of IgE, tryptase and eosinophils during the treatment with different dialysis membranes. Over time the skin rash disappeared with Vit-E dialyzer and tryptase concentration, eosinophil count and IgE levels decreased

A 73-year-old woman on chronic hemodialysis (HD) presented a relapsing itchy rash during her HD sessions (Fig. 1). She had been diagnosed with chronic kidney disease (CKD) 7 years earlier, in 2014, presumably due to oxalate nephropathy in enteric hyperoxaluria. At the time of diagnosis, she presented with radiological signs of chronic nephropathy with kidney stones and high urinary oxalate values. A genetic test ruled out primary hyperoxaluria. She started twice-weekly HD treatment in September 2019. In April 2021, she presented an itchy rash on her right ankle during a dialysis session, that immediately disappeared after discontinuing treatment. Recurrence of the rash was observed in the following HD sessions and it extended to all body areas followed by an intractable itch, unresponsive to antihistamines and intravenous steroids. Hemodynamic parameters were stable and no respiratory symptoms were observed. Biochemical parameters were not remarkably altered (PTH, calcium, phosphorus, urea, B2-microglobulin) and an immunologic panel was negative. All intra-dialytic pharmacological therapy was discontinued (iron carboxymaltose, low-weight molecular heparin and erythropoietin) without any improvement. Hypothesizing a type of urticaria of cholinergic origin the dialysate temperature was reduced from 36.5 to 35 °C, but there was no remission. Not even the use of the Bellco Flexia temperature biofeedback system during treatment prevented the rash.

The presumptive diagnosis was  a reaction to the low-flux polysulfone membrane dialyzer (Fresenius FX-10). Firstly, we changed the dialyzer using PMMA membranes (Toray Filtryzer-B3A and Filtryzer BG 2.1), based on the histamine-adsorbent property of this material, which resulted in a delay in the appearance of the symptoms, prolonged dialysis duration but no complete remission. We then used the AN69 (Baxter Evodial 1.6) and ATA membranes (Nipro Solacea 21H) with no improvement in the rash during dialysis treatment (Table 1).

**Table 1:** Main characteristics of the membranes used

Dialyzer membrane	Technique	Low vs High Flux	Sterilization	N. of treatments
PS	HD	LOW FLUX	Gamma	156
PMMA (B3A)	HD	LOW FLUX	Gamma	18
PMMA (BGU)	HD	HIGH FLUX	Gamma	5
AN69ST	HD-HDF	HIGH FLUX	Gamma	2
ATA	HD-HDF	HIGH FLUX	Gamma	2
EVAL	HD	LOW FLUX	Gamma	44
VIE-18	HD	HIGH FLUX	Gamma	Currently used

*PS* polysulfone, *PMMA* polymethyl methacrylate, *ATA* asymmetric cellulose triacetate, *EVAL* ethylene vinyl alcohol

Moreover, the measurement of IgE, tryptase and eosinophils performed during the dialysis session (at the beginning, after 30 min and after 2 h) showed no significant incremental trend (Fig. 1).

The only significant improvement was observed with the use of an EVAL dialyzer (ASAHI—Kuraray KF201), a low flux synthetic membrane, with the peculiarity of being intrinsically hydrophilic. Since this type of membrane was no longer in production, once our supply ran out, we chose a new polysulfone membrane coated with Vitamin E (ASAHI—ViE18) as an alternative, trusting that the Vitamin E coating might be a barrier between the blood and the polysulfone membrane (Table 1). These dialyzers were effective in preventing the rash from recurring, giving the patient the possibility to complete the dialysis session with no symptoms.

## Lesson for the clinical nephrologist

An immunologic interaction between blood cells and the artificial material occurs during every HD session. In 1988, Daugirdas characterized hypersensitivity hemodialysis reactions as type A (anaphylactic or anaphylactoid) and type B (non-specific). Type A reactions typically occur within the first 30 min: signs and symptoms may include urticaria, coughing, rhinorrhea, lacrimation, abdominal cramps, pruritus, a burning sensation, angioedema, dyspnea and even circulatory collapse and death. Type A reactions can be anaphylactic (IgE-mediated) or anaphylactoid (not-IgE mediated). Type B reactions are more common and have milder symptoms, which may include chest and back pain, nausea, and vomiting. They are considered secondary to the release of histamine, leukotrienes, and bradykinin [1].

Numerous attempts to improve the biocompatibility of membrane composition, sterilization methods and other materials in the dialyzers and tubing have been made. Nonetheless, the incidence of hypersensitivity reactions in the context of HD treatment is quite stable. Several cases of reactions to polysulfone membranes have been reported. In 2014, clinical reports were published regarding 7 patients with hypersensitivity reactions to synthetic dialysis membranes; most of them met the criteria for type A reaction [2]. Patients can be sensitive to synthetic membranes of different compositions, although in most cases membranes containing polyvinylpyrrolidone (PVP) were used [3]. There is controversy around the role PVP plays in triggering hypersensitivity reactions. PVP is the most commonly used hydrophilic additive that is blended to polysulfone to alter its hydrophobic features. Moreover, molecules such as glue, rubber and plastics can be responsible for a number of hypersensitivity reactions, as can heparin administered as an anticoagulant during dialysis [4]. Even surface characteristics of dialysis membranes (i.e., surface, electric charge) and dialyzer features may influence these biologic reactions. The use of PVP also increases the negative charge density and can result in activation of the Hageman factor and consequent overproduction of bradykinin [2].

In 2018, a multicenter Spanish study evaluated 1561 patients on maintenance HD and reported an incidence of 2.3% of hypersensitivity reactions. The membranes involved were polysulfone, polyethersulfone and polyacrylonitrile. However, at the time they concluded that the increase in published cases related to polysulfone membranes did not appear to correspond to a real increase in cases, considering that polysulfone was the most widely used membrane. The clinical pattern was neither clear nor easily recognizable, but symptoms disappeared in almost all cases after switching to another membrane, especially cellulose triacetate membranes [5]. Other studies confirmed that the majority of patients with hypersensitivity reactions to synthetic membranes demonstrated tolerance to cellulose triacetate membranes, with complete resolution of symptoms, since they do not contain Bisphenol A, a highly sensitizing compound [3].

Markers of interest for identifying hypersensitivity reactions include eosinophil count, tryptase, IgE, and complement fractions C3 and C4. However, as they can be normal or slightly increased, other elements such as timing, elimination of the offending agents, and resolution of clinical symptoms are truly important for diagnosis.

Management of hypersensitivity reaction is based on the discontinuation of the offending agent. In case of a life-threatening reaction, dialysis treatment should be stopped immediately without returning blood from the extracorporeal circuit back into the patient, and the administration of fluids, epinephrine, corticosteroids, and antihistamine should be considered [6].

In our case, the patient did not develop a life-threatening reaction, but she experienced an unbearable itchy rash which was unresponsive to steroids or antihistamines, thereby requiring the interruption of HD sessions. A beneficial effect was achieved by using an EVAL dialyzer, a symmetric, homogeneous membrane with hydrophilic segments that attract water thus creating a “dynamic water structure” on the membrane surface: this is believed to reduce blood-membrane interactions. Thanks to the hydrophilic segments, the membrane does not need any hydrophilic additives, such as PVP [7]. Dialysis using an EVAL membrane results in minimal platelet and coagulation activation and low IL-6 expression. The reduced interactions between blood and membrane surface were likely the key element that led to the complete resolution of the rash in our patient.

When our supply of EVAL dialyzers ran out, we replaced them with a new polysulfone vitamin-E coated membrane so as to limit the blood—dialyzer interaction, hoping that the presence of a vitamin-E layer might reduce the interaction between the patient’s blood and the polysulfone membrane. Vitamin E-modified polysulfone dialyzers were originally proposed due to their antioxidative and anti-inflammatory activities, in an effort to improve biocompatibility and protect chronic HD patients against the complications associated with long-term hemodialysis. The form of vitamin E used is alpha tocopherol since it has the highest physiological activity [7]. It is the sole biologically relevant free radical scavenger working in cooperation with a series of glutathione and thioredoxin-dependent peroxides expressed in tissues and biological fluids [8]. In the last decades, two systematic reviews on the effects of vitamin E-coated membranes have been published [7, 8]. The Authors demonstrated a positive effect of vitamin E-membranes on oxidative stress and inflammatory status. Indeed, a significant decrease in interleukin-6 levels and other inflammatory markers was observed. On the other hand, the use of vitamin E-membranes did not influence other parameters such as dialysis adequacy, lipid profile, or intradialytic blood pressure [9].

In conclusion, our patient presented a history of hypersensitivity reaction to HD membranes that could be classified as type A according to Daugirdas’ criteria. Unlike the majority of other cases, her symptoms resolved only with the use of a synthetic polysulfone membrane coated with Vitamin E, that reduced the interactions between the membrane polysulfone-PVP surface and blood, which proved to be a valid alternative, and may be suggested in similar cases.

## Data Availability

The datasets generated and/or analyzed during the current study are available from the corresponding author on reasonable request.
